# SSX2 is a novel DNA-binding protein that antagonizes polycomb group body formation and gene repression

**DOI:** 10.1093/nar/gku852

**Published:** 2014-09-23

**Authors:** Morten Frier Gjerstorff, Mette Marie Relster, Katrine Buch Viden Greve, Jesper Bonnet Moeller, Daniel Elias, Jonas Nørrelund Lindgreen, Steffen Schmidt, Jan Mollenhauer, Bjørn Voldborg, Christina Bøg Pedersen, Nadine Heidi Brückmann, Niels Erik Møllegaard, Henrik Jørn Ditzel

**Affiliations:** 1 Department of Cancer and Inflammation Research, University of Southern Denmark, Odense, DK-5000, Denmark; 1 Department of Cancer and Inflammation Research, University of Southern Denmark, Odense, DK-5000, Denmark; 1 Department of Cancer and Inflammation Research, University of Southern Denmark, Odense, DK-5000, Denmark; 2 Department of Cardiovascular and Renal Research, University of Southern Denmark, Odense, DK-5000, Denmark; 1 Department of Cancer and Inflammation Research, University of Southern Denmark, Odense, DK-5000, Denmark; 1 Department of Cancer and Inflammation Research, University of Southern Denmark, Odense, DK-5000, Denmark; 1 Department of Cancer and Inflammation Research, University of Southern Denmark, Odense, DK-5000, Denmark; 3 The Lundbeckfonden Center of Excellence NanoCAN, University of Southern Denmark, Odense, DK-5000, Denmark; 1 Department of Cancer and Inflammation Research, University of Southern Denmark, Odense, DK-5000, Denmark; 3 The Lundbeckfonden Center of Excellence NanoCAN, University of Southern Denmark, Odense, DK-5000, Denmark; 4 The Novo Nordisk Foundation Center for Protein Research, University of Copenhagen, Copenhagen, DK-2200, Denmark; 1 Department of Cancer and Inflammation Research, University of Southern Denmark, Odense, DK-5000, Denmark; 1 Department of Cancer and Inflammation Research, University of Southern Denmark, Odense, DK-5000, Denmark; 5 Department of Cellular and Molecular Medicine, University of Copenhagen, DK-2200, Denmark; 1 Department of Cancer and Inflammation Research, University of Southern Denmark, Odense, DK-5000, Denmark; 3 The Lundbeckfonden Center of Excellence NanoCAN, University of Southern Denmark, Odense, DK-5000, Denmark; 6 Department of Oncology, Odense University Hospital, Odense, DK-5000, Denmark

## Abstract

Polycomb group (PcG) complexes regulate cellular identity through epigenetic programming of chromatin. Here, we show that SSX2, a germline-specific protein ectopically expressed in melanoma and other types of human cancers, is a chromatin-associated protein that antagonizes BMI1 and EZH2 PcG body formation and derepresses PcG target genes. SSX2 further negatively regulates the level of the PcG-associated histone mark H3K27me3 in melanoma cells, and there is a clear inverse correlation between SSX2/3 expression and H3K27me3 in spermatogenesis. However, SSX2 does not affect the overall composition and stability of PcG complexes, and there is no direct concordance between SSX2 and BMI1/H3K27me3 presence at regulated genes. This suggests that SSX2 antagonizes PcG function through an indirect mechanism, such as modulation of chromatin structure. SSX2 binds double-stranded DNA in a sequence non-specific manner in agreement with the observed widespread association with chromatin. Our results implicate SSX2 in regulation of chromatin structure and function.

## INTRODUCTION

Polycomb group (PcG) proteins are negative regulators of gene expression essential for the maintenance of important biological processes such as cell identity, stem cell self-renewal and cell cycle regulation (1). PcG subunits are classically divided into two evolutionarily conserved complexes: Polycomb Repressive Complex 1 (PRC1) and Polycomb Repressive Complex 2 (PRC2), which act in concert or individually to control multiple cellular functions (2). PRC2 consists of the core subunits EED, EZH2 and SUZ12 and catalyzes tri-methylation of histone H3 at lysine 27 (H3K27me3). This modification serves as a platform for chromatin binding of PRC1, whereby the essential subunits BMI1 and RING1A/B promote mono-ubiquitination of histone H2A at lysine 119 (H2AK119ub). The H2AK119ub modifications are highly abundant and aid chromatin compaction (3). Although this model of ordered recruitment plays an essential role in PcG protein function, partial uncoupling of PcG proteins and H3K27me3, as shown by chromatin immunoprecipitation (ChIP) experiments, and different phenotypic effects caused by perturbing individual PcG members, suggest a highly complex function of these proteins (4). Furthermore, context-specific interactions with proteins and RNA molecules may increase the complexity of PcG protein function.

There is a strong link between dysregulation of PcG proteins and cancer development (1). BMI1 and EZH2 levels are elevated in several types of cancer (5–8) and there is increasing evidence that they promote tumor progression by supporting cellular self-renewal and proliferation, while blocking differentiation (9–15). PcG-mediated silencing of important tumor suppressor genes, including the INK4B-ARF-INK4A locus, partially enables this function (16–18). In addition, PcG proteins influence other cellular mechanisms central for tumor development, including apoptosis and the DNA damage response (19–25). Little is known about the role of PcG proteins in melanoma, but BMI1 and EZH2 are also deregulated and correlate with disease progression (5,26–28).

We describe herein a role for the germline- and cancer-associated protein SSX2 in modulation of PcG protein function. SSX proteins were first discovered as part of the fusion oncogene SYT-SSX (containing the 78 C-terminal amino acids of SSX1 or SSX2) (29), which plays an essential role in the development and progression of synovial sarcoma (30–32). SYT-SSX modulates chromatin in sarcoma cells through several mechanisms (30,32–35), one being its co-localization with PRC1 proteins and antagonization of PcG repressive function (36,37). An intimate relationship between SYT-SSX and PcG protein function is further emphasized by a recent study demonstrating that SYT-SSX associates with H3K27me3-modified chromatin (38). Many of these functions of SYT-SSX have been assigned to the SYT part of the fusion protein and little is known about the function of wild-type SSX. Members of the SSX family are restricted to cells at the spermatogonial and spermatocyte stage of spermatogenesis, but ectopic expression is seen in different types of tumors, including ∼30% of melanomas (39). The role of SSX2 in spermatogenesis and cancer development has remained elusive, but we demonstrate that SSX2 regulates PcG activity.

## MATERIALS AND METHODS

### Cell lines

A375 and MCF7 cells were obtained from American Type Culture Collection (which uses short tandem repeat profiling for cell line authentication). Clones with tetracycline (TET)-inducible expression of SSX2 (NM_175698) (A375-TET-SSX2) were generated using a modified Flp-In system (Invitrogen, Naerum, Denmark) with a TET-inducible cytomegalovirus promoter. In brief, A375 or MCF7 cells carrying a Flp recombinase recognition site were transfected with the pOG44 plasmid encoding the Flp recombinase and an expression vector with Flp recombinase recognition site, carrying the SSX2 cassette. Cells with stable integration of SSX2 were selected with 100–300-μg/ml hygromycin. Three independent clones with similar growth rates were pooled to avoid undesired effects of clonal selection. FM melanoma cells lines were originally established by A. Kirkin (40) and kindly donated by Professor MH Andersen, Center for Cancer Immunotherapy, Herlev Hospital, Denmark. Cells were grown in Dulbecco's modified Eagle's medium (A375, MCF7, HEK293) or RPMI (other melanoma cell lines) supplemented with 10% fetal bovine serum (FBS; Invitrogen), penicillin (100 U/ml) and streptomycin (100 mg/ml). Telomerase-immortalized human mesenchymal stem cells (hMSC-TERT20) were kindly provided by Professor M. Kassem, Department of Medical Endocrinology, Odense University Hospital, and grown in MEM, supplemented with 10% FBS (Invitrogen), penicillin (100 U/ml) and streptomycin (100 mg/ml). All cell lines were kept at low passage and cultured for no more than 3 months.

### Lentiviral transfections

Lentiviral particles with SSX2-specific shRNAs were purchased from Santa Cruz Biotech, Heidelberg, Germany (target sequences: 5′-GUA UGA GGC UAU GAC UAA A -3′ and 5′-GUU AGC GUU UAC GUU GUA U-3′). Cells were seeded at a density of 20 000 cells/cm^2^ and the next day transduced in media with 5 μg/ml of polybrene. After 16 h, media was changed and after another 48 h 0.2 μg/ml of puromycin was added to select stable transfectants. Cells were used for experiments after three passages in selective media.

### Transient transfections with SSX2-GFP

The GFP-SSX2 expression vector was made by inserting the SSX2 transcript variant 2 (NM_175698) open reading frame into pcDNA6.2-N-emGFP (Invitrogen). The resulting vector was transfected into A375 melanoma cells using TurboFect *in vitro* Transfection Reagent (Fermentas, Slangerup, Denmark) as described by the manufacturer.

### Salt and nuclease extraction of nuclei

To isolate nuclei, cells were incubated for 10 min in 10-mM HEPES (pH 7.9), 340-mM sucrose, 3-mM CaCl_2_, 2-mM MgAc, 0.1-mM ethylenediaminetetraacetic acid (EDTA) with Complete protease inhibitors (Roche). Pelleted nuclei were then sequentially incubated for 20 min in 20-mM HEPES (pH 7.9), 3-mM EDTA, 10% glycerol, 1.5-mM MgCl_2_ and Complete protease inhibitors (Roche, Hvidovre, Denmark) with 150-, 250- and 400-mM NaCl. The final extraction step was carried out in 150-mM HEPES (pH 7.9), 1.5-mM MgCl_2_, 150-m MgAc, Complete protease inhibitors (Roche) and 0.5-u/μl Benzonase (Sigma, Brondby, Denmark) for 20 min.

### Size-exclusion chromatography

A375-TET-SSX2 cells were grown with and without 50-ng/ml DOX for 24 h and harvested in phosphate buffered saline. Cells were incubated for 10 min in 10-mM HEPES (pH 7.9), 0.34-M sucrose, 3-mM CaCl_2_, 2-mM MgAc, 0.1-mM EDTA, Complete protease inhibitors (Roche) and 0.5% NP40 to release nuclei. Pelleted nuclei were extracted in 50-mM Tris-HCl (pH. 7.2), 300-mM NaCl, 0.5% NP40/Igepal, 1-mM EDTA, Complete protease inhibitors, using brief sonication and incubation on ice, and after centrifugation extracts were loaded on a Superose 12 column. Fractions were collected and analyzed by western blotting.

### DNA expression profiling

A375 cells with DOX-inducible expression of SSX2 (A375-TET-SSX2) cells were cultured with and without 50-ng/ml DOX for 48 h. Total RNA was purified, using Trizol (Invitrogen) according to manufacturer's recommendations, but with 1-bromo-3-chloropropane (BCP) instead of chloroform and repeated phenol-chloroform extractions. Gene expression analysis was conducted using Human Gene 1.0 ST Arrays (Affymetrix, CA, USA) in accordance with the recommendations of the manufacturer. The statistical analysis was conducted using Partek Genomic suite software in which the raw Affymetrix intensity measurements of all probe sets were background corrected, normalized and summarized into gene expression level measurements using Robust Multi-array Average (RMA). A one-way ANOVA was conducted to identify any difference between the groups, and later a stepwise comparison of the groups was made using the *t*-test. False discovery rate (FDR) of 0.05 was set to identify genes that were differentially expressed. Genes with an FDR of less than or equal to 0.05 and a fold change of at least 2 were classified as differentially expressed.

### Quantitative reverse transcriptase-polymerase chain reaction

Total RNA was purified using Trizol (Invitrogen) for tumor samples or the RNeasy® Mini Kit from Qiagen, Copenhagen, Denmark for cell culture samples. A RevertAid Premium Reverse Transcriptase kit (Fermentas) was used for cDNA synthesis. Relative quantification of gene expression was performed with SYBR green polymerase chain reaction (PCR) Mastermix (Applied Biosystems, Nearum, Denmark). Primers: BMP2 (Qiagen, QT00012544), SERPINB2 (Qiagen, QT00077497), ATF3 (Qiagen, QT00000273), PUM1 (Qiagen, QT00029421), SLC14A (Qiagen, QT01816514), TMEM27 (Qiagen, QT00030772), AREG (Qiagen, QT00030772), GAPDH (Qiagen, QT), beta-Actin (Qiagen, QT) and SSX-pan (5′-AGA AGC CAG CAG AGG-3′ and 5′-TGG GTC CAG GCA TGT-3′).

### Antibodies

Anti-beta-Actin (clone AC15, Abcam, Cambridge, UK; used in 1:5000 for Western Blotting (WB)), anti-SSX2/SSX3 (clone 1A4, Novus Biologicals, CO, USA; used 1:700 for IHC (immunohistochemistry), 1:100 for fluorescence microscopy and 1:100 for ICC (immunocytochemistry)), anti-SSX2-4 (clone E3 (39); used 1:3000 for WB), anti-BMI1 (Cell Signaling, MA, USA; used 1:200 for ICC, 1:100 for fluorescence IHC and 1:1000 for WB), anti-EZH2 (Cell Signaling; used 1:200 for ICC and 1:1000 for WB), anti-histone H3K27me3 (Cell Signaling; used 1:200 for ICC and 1:1000 for WB) and anti-H3 (clone FL136, Santa Cruz Biotech; used 1/1000 for WB).

### Immunostaining

Methods for immunocyto- and IHC and western blotting were described previously (41).

### Polycomb target gene quantitative PCR

SSX2-specific shRNA vector (#20147, Sigma) or vector-only (pLKO1) lentiviruses were prepared using standard methods. In brief, HEK293T cells were co-transfected with shRNA vector and packaging plasmids pVSVG, pRSV-Rev and pMDL g/p RRE (plasmids were kindly provided by the Tronolab through Addgene, Cambridge, MA, USA), and after 72 h, supernatant containing virus was harvested and transferred to FM45 target cells. Forty-eight hours after transduction, FM45 cells were harvested and RNA isolated, as described above. Preparation of cDNA and gene expression analysis, using the Human Polycomb and Trithorax genes RT2 profiler PCR array, was carried out as recommend by the manufacturer (Qiagen).

### Expression and purification of recombinant SSX2

SSX2 was expressed in *Escherichia coli* and purified by affinity and size-exclusion chromatography (details are available upon request).

### ChIP analysis

A375-TET-SSX2 cells were grown with 50-ng/ml DOX for 24 h to induce SSX2 expression. ChIP was done using the Simple Chip Chromatin IP kit (Cell Signaling) according to the recommendations of the manufacturer. Experiments were run in duplicate and repeated twice. Potential SSX2, BMI1 and H3K27me3 binding sites within promoters and transcription start sites of *ATF3* (Chr1:212782257-212792953), *SERPINB2* (Chr18:63887705-63903890) and *TMEM27* (chrX: 15627316-15665031) genes were selected with the UCSC genome browser. ChIP-PCR primers are provided in Supplementary Information.

### Electrophoretic mobility shift assays

Electrophoretic mobility shift assays were done by incubating ∼10-pg double-stranded ^32^P-labeled DNA fragments with recombinant SSX2 (rSSX2; 0–200 ng/μl) in 10-μl binding buffer (20-mM Tris-HCl, 50-mM NaCl pH 7.4, 1-mM DTT). Following electrophoresis, the SSX2–DNA complexes were detected by autoradiography, or using phosphor imager storage screens. The DNA fragments were a 181-bp HindIII-PvuII fragment from pUC19 or a 307-bp EcoRI-PvuII fragment from pXen/pUC19. pXen/pUC19 was constructed by inserting the sequence 5′-GAT CCA CCT GGT ATT CCC AGG CGG TCT CCC ATC CAA GTA CTA ACC AGG CCC GAC CCT GCT TGG CTT CCG AGA TCA -3′ in the BamH1 site of pUC19.

### Microscale thermophoresis

Serial dilutions of rSSX2 or bovine serum albumin was incubated with 50 nM of a Cy5-labeled random 30-nt double-stranded DNA (dsDNA) (5′-CGT CTA TAC TTT CAC AAT GTT GAC CTG CAC-3′) in 20-mM Tris-HCl, 50-nM NaCl (pH 7.4), 0.05% NP40 at ambient temperature for 2 h. Microscale thermophoresis measurements were performed utilizing a Monolith NT.115 instrument (NanoTemper Technologies GmbH, Munchen, Germany). The measurements were performed using standard capillaries at 30% LED excitation and 80% MST power, with a laser-on time of 30 s and a laser-off time of 5 s. NanoTemper Analysis 1.2.20 software was used to fit the data and to determine the *K*_d_.

### Statistics

The non-parametric Mann Whitney test was chosen in the experiments involving the number of γH2AX, Bmi1 and EZH2 bodies, where normality cannot be assumed. Student's *t*-test or *Z*-test was used in all other experiments.

## RESULTS

### Ectopically expressed SSX2 disintegrates BMI1 and EZH2 nuclear bodies and induces the expression of PcG complex target genes

**Figure 1.**
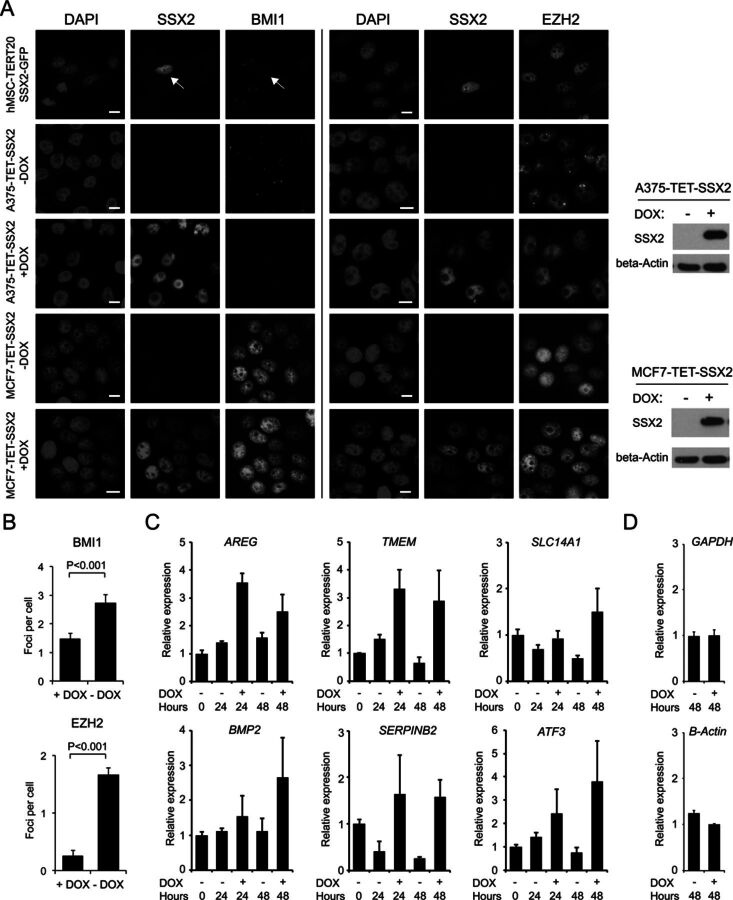
Ectopically expressed SSX2 disintegrates PcG nuclear bodies and derepresses PcG-regulated genes. (**A**) SSX2-GFP was transiently expressed in human mesenchymal stem cells (hMSC-TERT20) and SSX2 was expressed in A375 melanoma cells and MCF7 breast cancer cells using a DOX-inducible system (50-ng/ml DOX; western blots showing SSX2 levels inserted). Cells were fixed and stained for SSX2, BMI1 and EZH2 48 h after transfection/DOX treatment. (**B**) The number of BMI1 and EZH2 nuclear bodies in A375 cells with or without SSX2 expression was quantified from at least three individual frames of cells (*N* > 70). A Mann Whitney test was used for statistical analysis. Error bars indicate standard errors of means. (**C, D**) The expression of PcG target genes (C) and non-PcG target genes (D) in A375 cells was quantified using qPCR at 24 and 48 h after DOX induction (50 ng/ml) of SSX2 expression. A two-tailed student's *t*-test was used for statistical analysis. Data represent the mean ± SD for three biological replicates.

**Figure 2.**
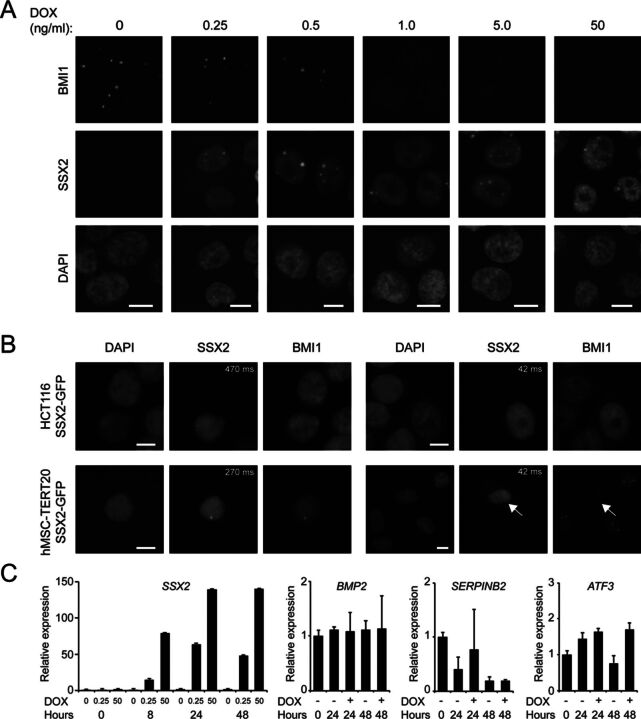
SSX2 modulates PcG nuclear bodies in a dose-dependent manner. (**A**) The level of SSX2 expression in A375 cells was titrated using increasing concentrations of DOX and after 24 h the cells were fixed and stained for SSX2 and BMI1. (**B**) SSX2-GFP was transiently expressed in HCT116 and hMSC-TERT20 cells and after 24 h the cells were fixed and stained for BMI1. Short and long exposure times were used for cells with high and low SSX2-GFP expression, respectively. (**C**) qPCR analysis of the expression of SSX2 at increasing DOX concentrations, and PcG target genes at 0.25-ng/ml DOX, in A375 cells. Error bars indicate standard deviations. Scale bars = 5 μm. Data represent the mean ± SD for three biological replicates.

Previous studies have shown that the SYT-SSX fusion protein co-localizes with PcG nuclear bodies in sarcoma cell lines and derepresses PcG target gene expression (37). To investigate the role of wild-type SSX2 in the regulation of PcG protein function, we expressed the protein in SSX-negative cancer cell lines derived from tumor types that frequently express SSX2 (39,42–44), including melanoma (A375; DOX inducible model), breast cancer (MCF7; DOX inducible model), colon cancer (HCT116) and tumorigenic mesenchymal stem cells (hMSC-TERT20; sarcomas may be derived from transformed hMSC (45)). The subcellular localization of SSX2, BMI1 (PRC1) and EZH2 (PRC2) in these cell lines was examined using immunofluorescence. We found that while hMSC-TERT20, HCT116 and A375 cells contained distinct nuclear BMI1 bodies, MCF7 cells exhibited a more diffuse nuclear distribution of BMI1 (Figures 1A and B and 2B). Interestingly, ectopic expression of SSX2 changed the nuclear localization pattern of BMI1 from focal to diffuse in hMSC-TERT20 and A375 cells. EZH2 bodies (PRC2) were only present in A375 cells and were lost upon ectopic SSX2 expression (Figure 1A and B). These results demonstrate that SSX2 is able to disintegrate PcG nuclear bodies in human cancer cells.

To investigate whether the SSX2-mediated disintegration of BMI1 and EZH2 nuclear bodies in A375 melanoma cells influenced the repressive function of these proteins, we performed global gene expression profiling of A375 cells with and without ectopic SSX2 expression. This analysis showed that the expression of several known PcG target genes was induced by SSX2, including *AREG, TMEM, SLC14A1, BMP2, SERPINB2* and *ATF3* (Supplementary Table S1). The expression of these six PcG target genes was confirmed by quantitative PCR (qPCR) to be upregulated by SSX2 (Figure 1C), while two non-PcG target genes (i.e. *GAPDH* and *beta-Actin*) were unaffected (Figure 1D).

### The effect of SSX2 on PcG nuclear bodies is concentration dependent

We used increasing concentrations of DOX to induce different levels of SSX2 expression in the A375 cells and evaluated the presence of BMI1 PcG bodies (Figure 2A). Surprisingly, we found that after 24 h low levels of SSX2 expression (induced by 0.25–0.5-ng/ml DOX) resulted in co-localization of SSX2 and BMI1 in nuclear bodies, whereas higher SSX2 levels (induced by 1–50-ng/ml DOX) promoted disintegration of BMI1 nuclear bodies. Similarly, co-localization of SSX2 and BMI1 in nuclear bodies was detected in hMSC-TERT20 and HCT116 cells with low SSX2-GFP expression, while cells with high expression of SSX2-GFP were devoid of BMI1 bodies (Figure 2B). These results indicate that a certain level of SSX2 is required to disintegrate PcG bodies. Some SSX2 nuclear bodies were BMI1-negative and some persisted after disruption of BMI1 bodies, demonstrating that SSX2 also exhibits BMI-1-independent chromatin binding (Figure 2A). Next, we examined whether co-localization of SSX2 and BMI1 was sufficient to derepress PcG-repressed genes in A375 cells (Figure 2C). Induction of low levels of SSX2 expression with 0.25-ng/ml DOX resulted in co-localization between SSX2 and BMI1 at 24 h, but this DOX concentration did not affect the expression of genes induced by high levels of SSX2 (i.e. *BMP2, SERPINB2* and *ATF3*). After 48-h incubation with 0.25-ng/ml DOX, BMI1 foci gradually disintegrated and *ATF3* expression was induced. These results demonstrate that PcG nuclear body disintegration is required for SSX2-mediated antagonization of PcG gene repression.

### Endogenous expression of SSX2 represses PcG body formation

**Figure 3.**
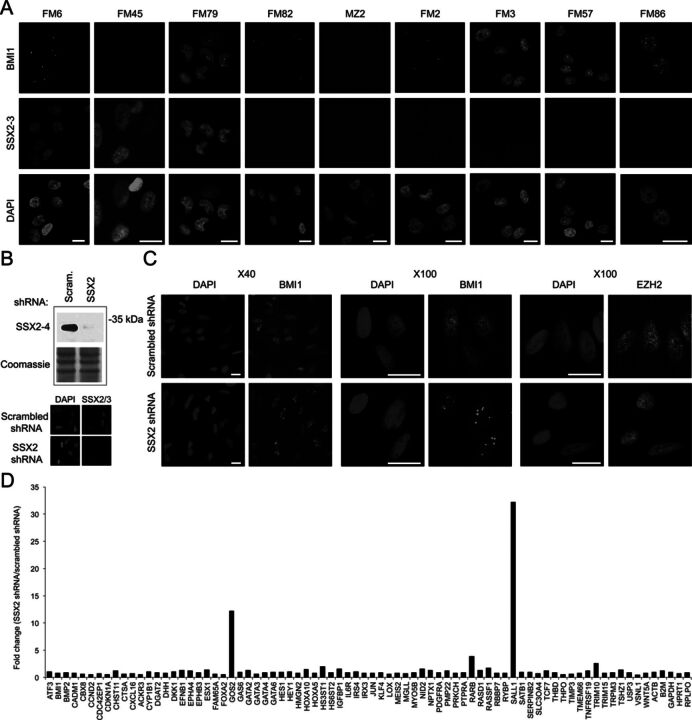
Endogenously expressed SSX2 represses PcG body formation. (**A**) The expression of SSX family members was investigated in a panel of melanoma cell lines using immunocytochemical staining (with the 1A4 antibody recognizing SSX2/3) and compared to the presence of PcG bodies. (**B**) The SSX2 expression in the FM45 cell line was knocked down using lentiviral transduction with shRNA vectors. A vector carrying a scrambled siRNA sequence was used as control. (**C**) FM45 cells with stable knockdown of SSX2 or with scrambled siRNA were fixed and stained for BMI1 and EZH2. Scale bars = 10 μm. (**D**) Expression of polycomb target genes in FM45 cells transduced with an SSX2-specific shRNA vector relative to cells transduced with empty vector.

SSX proteins are expressed in about 30% of melanomas (39) and having demonstrated that ectopic SSX2 expression regulated PcG nuclear body formation in A375 melanoma cells, we next investigated the effect of SSX2 on PcG function in melanoma cells with endogenous SSX expression. We correlated SSX expression with the presence of PcG nuclear bodies in a panel of melanoma cell lines (Supplementary Figure S1 and Figure 3A) and found that SSX2/3 (as determined by Mab E3 staining) was present in three of the nine cell lines (i.e. FM6, FM45 and FM79). However, other functionally redundant SSX genes may be differently expressed in the melanoma cell lines, as demonstrated by pan-SSX qPCR (Supplementary Figure S1). The presence of SSX2/3 did not exhibit a clear correlation with the absence of PcG nuclear bodies, as only one of the three SSX2/3-positive lines, i.e. FM45, was devoid of BMI1 nuclear bodies (Figure 3A). However, the FM45 melanoma cell line exhibited higher levels of SSX-pan expression than FM6 and FM79, as determined by qPCR (Supplementary Figure S1), further suggesting that SSX-mediated disintegration of PcG bodies is concentration dependent. In addition, there may be SSX-independent regulation mechanisms of PcG body formation.

Since FM45 melanoma cells are SSX2/3-positive and do not contain BMI1 PcG bodies, we investigated whether SSX2 is, in fact, responsible for the absence of BMI1 bodies in these cells. We knocked down the expression of SSX2 using lentiviral shRNA transfections and selected stably transfected cells with a strong reduction in SSX2 expression (Figure 3B). SSX2 knockdown was found to promote the formation of large BMI1 bodies in most cells (Figure 3C). We also examined the effect of SSX2 knockdown on the nuclear pattern of EZH2, but observed no change in nuclear localization pattern for this protein. We further examined the expression on a panel on known PcG target genes in FM45 cells with or without SSX2 expression (Supplementary Figure S2) and found that several genes exhibited increased expression in response to SSX2 knockdown (Figure 3D). This strongly indicates that endogenous SSX2 indeed inhibits BMI1 PcG body formation and repressive function in FM45 melanoma cells.

### SSX2 does not change the stability, structure or biochemical properties of PRC1 and PRC2

**Figure 4.**
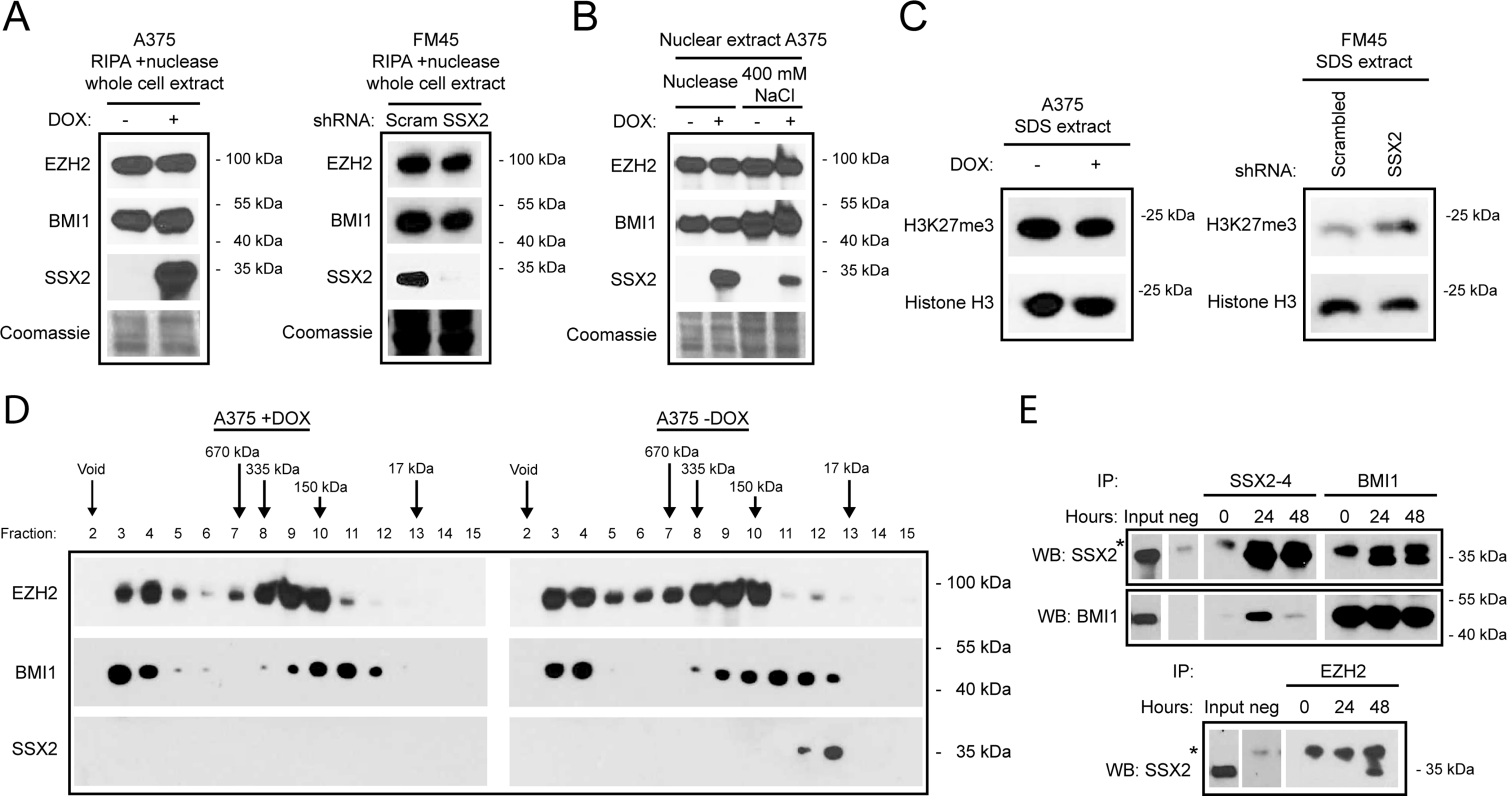
SSX2 does not change the level, biochemical properties or gene association of PcG proteins. (**A**) The levels of BMI1 and EZH2 were investigated in A375 cells with or without DOX-induced SSX2 expression, and in FM45 cells with or without knockdown of SSX2 expression. (**B**) The ‘extractability’ of BMI1, EZH2 and SSX2 was investigated in A375 cells with or without induced expression of SSX2, using western blotting of high salt and nuclease nuclear extracts. (**C**) The level of H3K27me3 was investigated in sodium dodecyl sulphate (SDS) buffer extractions of nuclear preparations of A375 cells with or without DOX-induced SSX2 expression, and of FM45 cells with or without knockdown of SSX2 expression. (**D**) The size of BMI1 and EZH2 nuclear complexes in high salt extracts from A375 cells, with or without induced expression of SSX2, was examined using size-exclusion chromatography and western blotting. (**E**) Co-immunoprecipitation of SSX2 with BMI1 or EZH2 from nuclease extracts of A375 cells with or without induced SSX2 expression. Fifty-ng/ml DOX was used in all experiments. *Non-SSX2 reactivity.

To further elucidate the mechanism of SSX2-mediated destabilization of PcG nuclear bodies, we investigated the stability of PRC1 and PRC2 core subunits (i.e. BMI1 and EZH2, respectively) in A375 cells with DOX-induced expression of SSX2 and in FM45 cells with knockdown of SSX2, and showed that SSX2 did not affect the expression levels of BMI1 and EZH2 (Figure 4A). We also investigated the extractability of BMI1 and EZH2 from nuclear preparations of A375 cells using high salt and nuclease, but SSX2 did not affect the properties of these proteins (Figure 4B). This suggested that the overall nuclear distribution (‘free’ protein versus ‘complex-bound’ and ‘chromatin-attached’ protein) of BMI1 and EZH2 was not altered as a consequence of SSX2 expression (Figure 4B).

Histone H3K27me3 is closely associated with PcG complex function, thus the effect of SSX2 knockdown on the level of this histone mark was investigated (Figure 4C). Ectopic expression of SSX2 in A375 melanoma cells did not affect the overall level of H3K27me3, but the level was, however, slightly increased in FM45 cells with reduced SSX2 expression. To investigate if the SSX2-mediated disruption of PcG nuclear bodies was due to a change in the structure of PRC1 and/or PRC2, we assessed the molecular size of protein complexes containing BMI1 and EZH2 in A375 cells with and without SSX2. Nuclear protein extracts analyzed by size-exclusion chromatography revealed the presence of ‘complex bound’ and ‘free’ BMI1 and EZH2 (Figure 4D) both in cells with and without SSX2. However, SSX2 did not change the relative distribution of ‘complex-bound’ and ‘free’ BMI1 and EZH2. These results suggest that SSX2 does not change the overall composition of PRC1 and PRC2.

Size-exclusion chromatography did not demonstrate any association between PcG complexes and SSX2. A potential interaction between chromatin-bound SSX2 and PcG complexes was further investigated using co-immunoprecipitation from nuclease-extracted A375 nuclei and demonstrated an interaction between SSX2 and both BMI1 and EZH2 (Figure 4E) that may reflect adjacent chromatin binding of the proteins due to incomplete chromatin digestion. The association with EZH2 was observed 48 h, but not 24 h, after induction of SSX2 expression, suggesting an interesting dynamic. This may reflect an SSX2-induced reorganization of chromatin. The apparent inconsistency between SSX2 and BMI1 immunoprecipitations at the 48-h time point may be due to structural changes affecting the binding of the SSX2 antibody.

### SSX2/3 correlates with distinct nuclear patterns of BMI1, EZH2 and H3K27me3 in spermatogenesis

**Figure 5.**
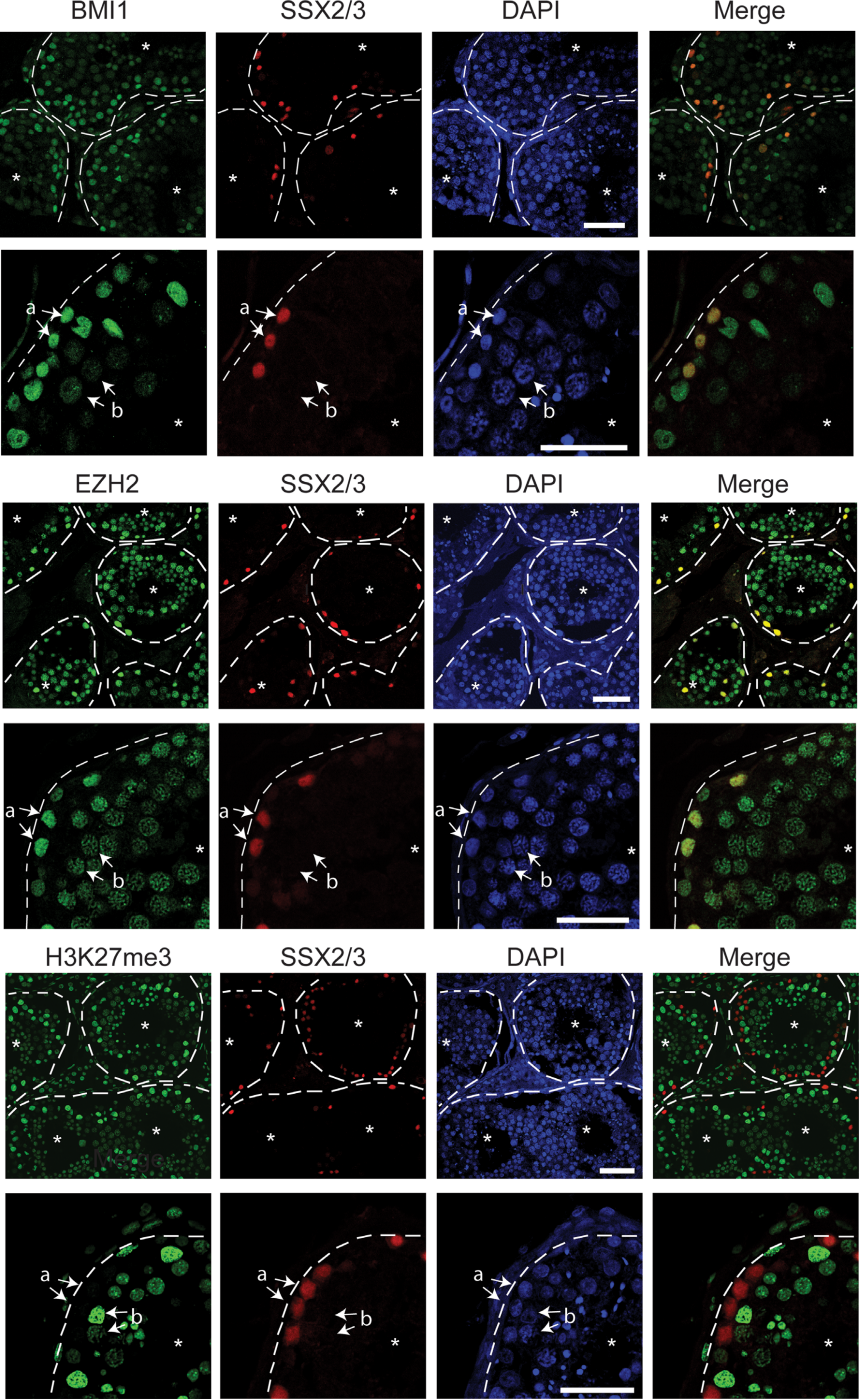
SSX2/3 negatively correlates with H3K37me3 in spermatogenesis. The expression patterns of BMI, EZH2 and H3K27me3 were compared to that of SSX2/3 in human testis tubules. Seminiferous tubules are indicated with dashed lines. Asterisks indicate the central lumen of tubules, ‘a’ indicates spermatogonia and ‘b’ indicates spermatocytes. Merges of red and green channels are shown. Scale bars = 50 μm.

As SSX proteins are naturally expressed at the spermatogonial stage of spermatogenesis (39), we investigated the expression patterns of SSX2/3, BMI1, EZH2 and H3K27me3 in testis tubules using immunohistochemical staining. This confirmed that SSX2/3 was strictly expressed in spermatogonia, and further revealed relatively high BMI1 and EZH2 expression in these cells (Figure 5, ‘a’ arrows) compared to the later stage spermatocytes (Figure 5, ‘b’ arrows). PcG nuclear bodies were not detected in spermatogonia or spermatocytes, suggesting a different chromatin organization in germ cells than somatic cells. The pattern of nuclear distribution of BMI1 and EZH2 changed from spermatogonia to spermatocytes, but this mirrored the change in chromatin structure (i.e. more condensed chromatin in spermatocytes). Interestingly, we found that spermatogonia were negative for H3K27me3, while this modification was highly present in the spermatocytes and later stages of spermatogenesis (Figure 5). These results demonstrate a clear inverse relationship between SSX2/3 expression and the presence of H3K27me3, but whether SSX2/3 is in fact involved in regulation of H3K27me3 formation in spermatogonia remains to be determined.

### SSX2 exhibits strong association with chromatin

**Figure 6.**
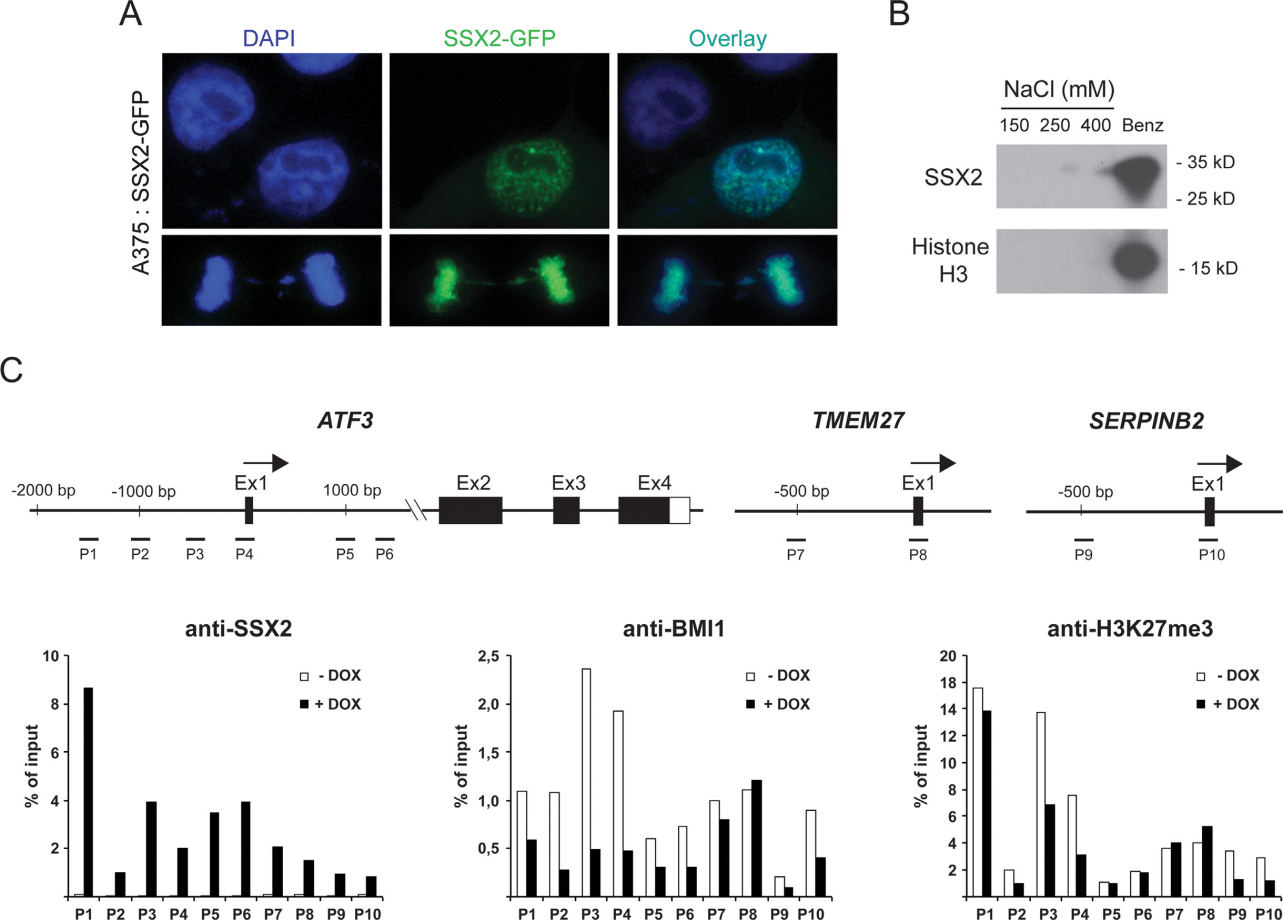
SSX2 exhibits strong association with chromatin. (**A**) A375 cells were transfected with SSX2-GFP and the chromatin association in interphase (top) and during mitosis (bottom; late anaphase) was investigated by ICC. (**B**) Salt and nuclease (Benzonase) extraction of SSX2 and Histone H3 from nuclear preparations of A375-TET-SSX2 cells with induced SSX2 expression (50 ng/ml). (**C**) ChIP-PCR analysis of SSX2, BMI1 and H3K27me3 association with the *ATF3, TMEM27 and SERPINB2* genes in A375-TET-SSX2 cells with or with DOX (50 ng/ml) induced expression of SSX2. Levels were normalized to input samples. Data represent the average of two biological replicates.

Staining of SSX2/3 in clinical specimens of melanoma demonstrated an association with chromatin that was confirmed by co-localization of SSX2 with chromatin in A375 melanoma cells during both inter- and metaphase (Figure 6A). In interphase cells, SSX2-GFP was distributed in multiple chromatin-associated bodies of different size, while the protein appeared to uniformly coat the condensed chromatin in mitotic cells. Salt and nuclease extraction of A375 cells with SSX2 expression further demonstrated that the chromatin association of SSX2 was resistant to high levels of NaCl (400 mM) and that nuclease treatment was needed to extract this protein. These results indicated that SSX2 exhibits a strong interaction with chromatin (Figure 6B). We next examined if derepression of the PcG target genes *ATF3, SERPINB2 and TMEM27* was associated with binding of SSX2 to the genes. We found extensive SSX2 binding to the proximal promoters and intron 1 of these genes (Figure 6C), consistent with the widespread binding of SSX2 to chromatin observed by microscopy. A reduction in BMI1 and H3K27me3 upon SSX2 binding was observed for *ATF3* and *SERPINB2*, but not for *TMEM27* (Figure 6C). Furthermore, there was a high degree of non-concordance between SSX2 and BMI1/H3K27me3 presence at *ATF3 and SERPINB2*. These results suggest that the antagonistic effect of SSX2 on PcG function is mediated through indirect mechanisms such as structural modulation of chromatin.

### SSX2 binds dsDNA in a sequence non-specific manner

**Figure 7.**
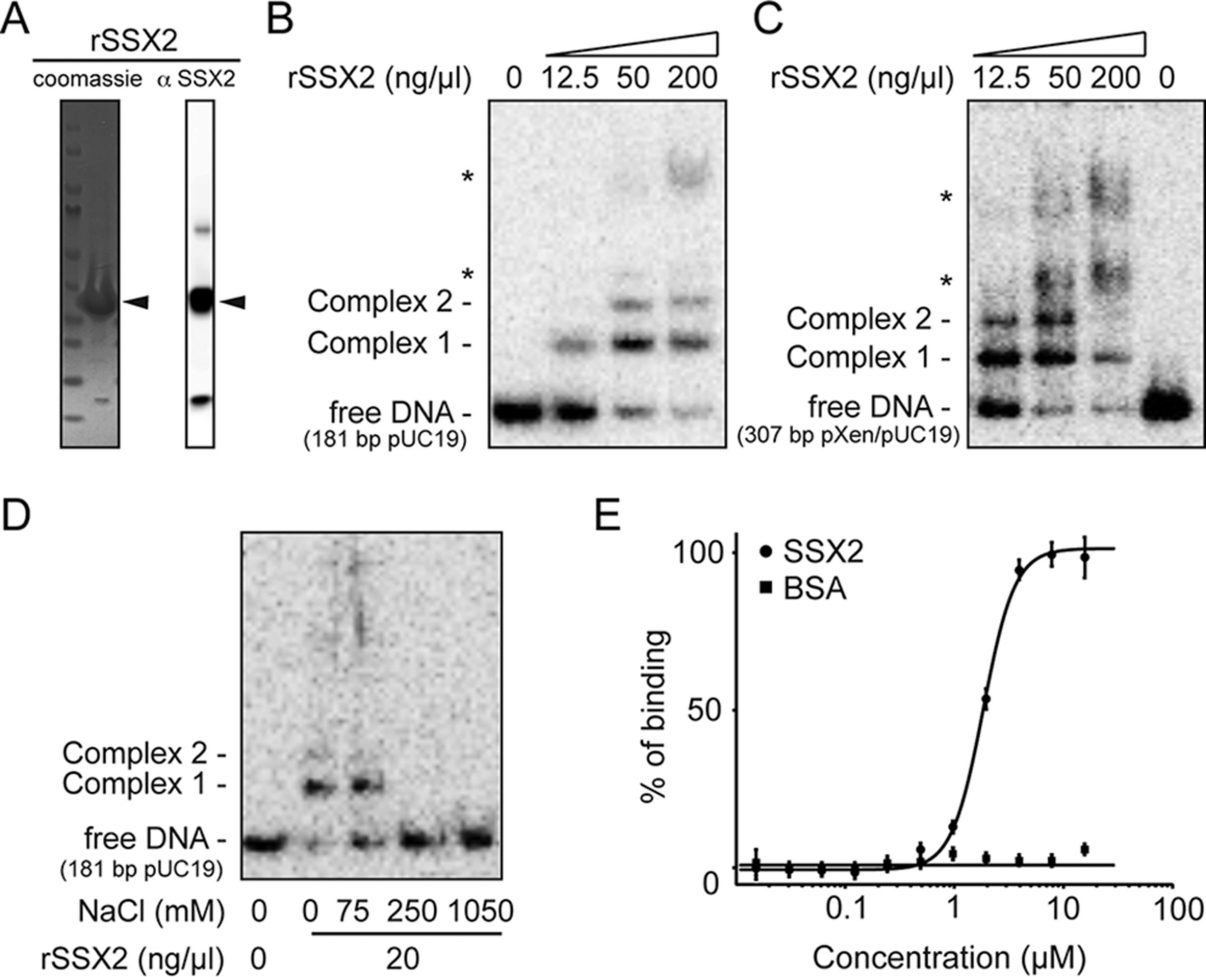
SSX2 binds dsDNA. (**A**) Purified rSSX2 was characterized by sodium dodecyl sulphate-polyacrylamide gel electrophoresis (SDS-PAGE) followed by Coomassie staining (25 μg) and western blotting (0.5 μg). (**B, C**) Electrophoretic mobility shift assay showing rSSX2 binding to 10 pg of two different ^32^P-labeled dsDNA restriction fragments. *Higher order complexes. (**D**) Electrophoretic mobility shift assay examining the salt stability of SSX–dsDNA complexes. Complexes were allowed to form before addition of NaCl. (**E**) Analysis of the rSSX2–dsDNA interactions by microscale thermophoresis. Cy5-labeled dsDNAs (30 bp, 50nM) were incubated with increasing concentrations of SSX2. Binding of SSX2 to dsDNA was plotted using the Hill equation, yielding a *K*_d_ of 1.8 ± 0.2 μM. Bovine serum albumin (BSA) was used as a negative control. Data represent the mean ± SEM for four technical triplicates. Binding percentage represents normalized fluorescence signals (amplitude 113.5).

Due to the lack of a canonical DNA-binding domain, SSX2 was previously suggested to interact indirectly with DNA through PcG proteins (37), but to our knowledge direct binding to DNA has never been tested. To investigate the ability of SSX2 to bind dsDNA, rSSX2 was used for electrophoretic mobility shift assays (Figure 7A–D). Two different, sequence-unrelated dsDNA restriction enzyme fragments formed discrete complexes with slower electrophoretic mobility upon incubation with rSSX2 (Figure 7B and C). Both dsDNA fragments bound rSSX2 with similar affinities and two distinct nucleoprotein complexes were formed (Figure 7B and C, ‘complex 1’ and ‘complex 2’), suggesting potential oligomerization of rSSX2. Diffuse higher order complexes also appeared at higher protein concentrations of rSSX2 (Figure 7B and C, asterisks). Complex 1 and complex 2 were both stable at 75-mM NaCl, but were dissolved at 250-mM NaCl (Figure 7D).

The binding of SSX2 to DNA was further analyzed by microscale thermophoresis. SSX2 exhibited binding to a fluorescently labeled 30-nt dsDNA with a *K*_d_ of 1.8 ± 0.2 μM. A curve was fitted to the data points using a Hill equation with a coefficient of 3.0, which may suggest positive cooperative binding. These data demonstrate that SSX2 can bind dsDNA directly *in vitro* in a sequence non-specific manner and suggest that SSX2 may modulate the structure of chromatin through direct binding to DNA.

## DISCUSSION

PcG proteins can be found in nuclear concentrations called PcG bodies, which may vary in size and number among different types of cells (46), and these structures appear to be important for PcG function. From *Drosophila*, there is evidence that long-range chromosomal interactions of PcG-repressed genes occur in PcG bodies (47–49). Furthermore, recent results suggest that chromatin insulators, not PcG response elements, are responsible for targeting PcG-repressed genes to PcG bodies (50). However, little is known about the composition and functional importance of these PcG bodies in healthy and diseased mammalian cells. Here we present evidence that the germline- and cancer-associated protein SSX2 interacts with PcG bodies leading to their disintegration and derepression of PcG target genes.

While we demonstrate that SSX2 antagonizes PcG function through disintegration of PcG bodies, our results further show that SSX2 does not affect the overall level or structure of BMI1- and EZH2-containing PcG complexes, nor does SSX2 seem to interact directly with PcG complexes. Conversely, SSX2 exhibits a strong association with chromatin and binds dsDNA directly *in vitro*. Thus it is likely that binding of SSX2 to dsDNA *in vivo* may change chromatin structure and organization, promoting loss of PcG complexes and H3K27me3 from repressed genes and a release of PcG target genes from the repressive environment of PcG bodies. SSX2 may accomplish this through direct structural modification of DNA, by changing the activity of regulators of chromatin structure (e.g. enhancers) and/or by directly reducing chromatin binding of PcG complexes to chromatin. The DNA-binding specificity of SSX2 and effect on higher order chromatin structure will be the subject of future studies, but our analysis of the target genes (i.e. *ATF3*, *TMEM27* and *SERPINB2*) provides some important information in this regard. SSX2 was observed to associate widely with the promoters, transcription start sites and intron 1 of these genes, suggesting that SSX2 has rather unselective DNA-binding properties. This is in accordance with microscopy analysis showing that SSX2 is widely distributed in the genome although accumulating in distinct (PcG and non-PcG) chromatin-associated foci in interphase cells. Analysis of target genes also suggested that SSX2 promotes loss of BMI1 and H3K27me3 from promoters and gene bodies. However, the high degree of non-concordance between SSX2 and BMI1/H3K27me3 presence suggests that SSX2 antagonizes PcG function indirectly through, for instance, structural modulation of chromatin. A role for SSX2 in inhibiting genome-wide H3K27me3 is further supported by increased levels of H3K27me3 upon SSX2 knockdown in melanoma cells and a clear inverse correlation between SSX2 and this histone mark in spermatogenesis. As H3K27me3 serves as a binding scaffold for PRC1 complexes, H3K27me3-loss and a subsequent reduction in PRC1 binding may be part of the mechanism of SSX2-mediated derepression of PcG target genes. In *Drosophila* terminal differential of testis germ cells involves antagonization of PcG gene repression (51). SSX2 may be involved in similar reprogramming of chromatin in human spermatogenesis.

Dysregulation of PcG protein activity is emerging as an important contributor to oncogenesis. Previous studies of PcG proteins in cancer have focused on changes in PcG levels during cancer progression (2), but the results presented herein demonstrate a novel level of PcG functional perturbation in cancer through SSX2-mediated control of polycomb body formation. As SSX2 is expressed in multiple types of human cancer, including 30% of melanomas, this protein may be an important player in the chromatin reprogramming instrumental for cancer development.

As shown in this study, wild-type SSX2 has overlapping functions with SYT-SSX2 as it interacts with PcG bodies and antagonizes PcG repressive function. However, there are also differences in the function of SSX2 and SYT-SSX2 in that only the latter has been shown to destabilize and reduce the overall levels of BMI1 in sarcoma cells (36), an effect not observed with SSX2 in melanoma cells. On the other hand, SSX2 disintegrated PcG nuclear bodies, an effect not described for SYT-SSX2, although it seems essential for SSX2-mediated antagonization of PcG repressive function. Differences between SSX2 and SYT-SSX functions may be assigned to the SYT domain of the latter. Our finding that SSX2 bind dsDNA may also be important for understanding SYT-SSX2. A DNA-binding activity of SYT has not been described and the protein contains no canonical DNA-binding motifs, thus SSX2 may be implicated in defining the DNA-binding properties of SYT-SSX2, although it is currently not known if the part of SSX2 present in SYT-SSX2 includes the DNA-binding domain.

In conclusion, our study demonstrates a role for SSX2 in regulation of PcG function with implications for our understanding of the processes of spermatogenesis and oncogenesis.

## Supplementary Material

Supplementary Data
