# Correction to ‘SSX2 is a novel DNA-binding protein that antagonizes polycomb group body formation and gene repression’

**DOI:** 10.1093/nar/gkag242

**Published:** 2026-03-14

This is a correction to: Morten Frier Gjerstorff, Mette Marie Relster, Katrine Buch Viden Greve, Jesper Bonnet Moeller, Daniel Elias, Jonas Nørrelund Lindgreen, Steffen Schmidt, Jan Mollenhauer, Bjørn Voldborg, Christina Bøg Pedersen, Nadine Heidi Brückmann, Niels Erik Møllegaard, Henrik Jørn Ditzel, SSX2 is a novel DNA-binding protein that antagonizes polycomb group body formation and gene repression, Nucleic Acids Research, Volume 42, Issue 18, 13 October 2014, Pages 11433–11 446, https://doi.org/10.1093/nar/gku852

In September 2025, concerns were raised on PubPeer (https://pubpeer.com/publications/3663D538A015178791D67F92D393A2#2) regarding the possible duplication of Coomassie gels in Figures 4A and 4B. The authors reviewed the issue and now wish to correct Figure 4.

The authors confirmed that incorrect lanes from the Coomassie staining were included during figure assembly. They have provided the journal with the original underlying data, which is included in the Supplementary Information accompanying this correction notice. Revised versions of Figures 4A and 4B are presented below.

This correction does not affect the results, discussion and conclusions presented in the article. The figure has been corrected only in this correction notice to preserve the published version of record.


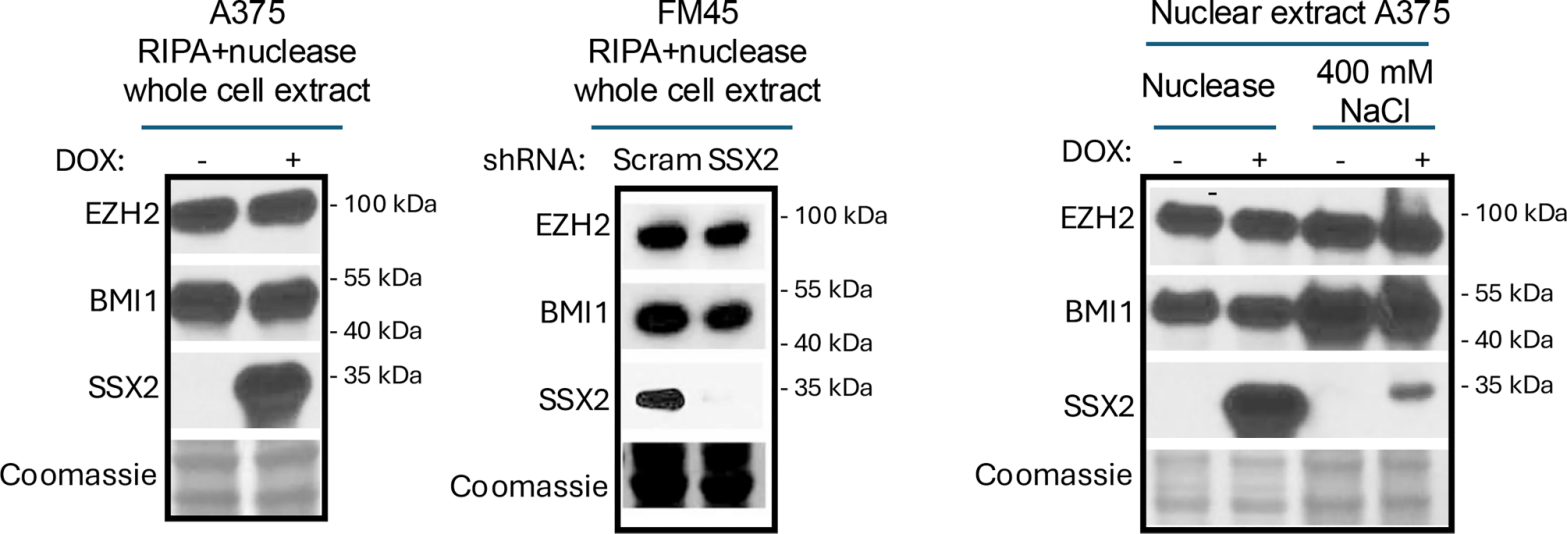


Corrected Figure 4A and 4B.

## Supplementary Material

gkag242_Supplemental_Files

