# Supplementary material for: SSX2 is a novel DNA-binding protein that antagonizes polycomb group body formation and gene repression
Source: Nucleic Acids Res. 2014 Sep 23;42(18):11433–46. doi: 10.1093/nar/gku852 (PMC4191419; doi:10.1093/nar/gku852)
Supplement: Supplementary Data [file nar_42_18_11433_s1.zip › nar-03727-x-2013-File009.pdf]

Supplementary Information

Primers for quantitative ChIP-PCR

ATF3-P1-F, 5’- AAT CGG TTC AGG TCC AGA GC -3’

ATF3-P1-R 5’- GGG TCT CAC TTG AAG TCC CC -3’

ATF3-P2-F, 5’- TGA GGG CAG AGG GGA TTT CT -3’

ATF3-P2-R, 5’- CCC CGC TCT CAG GCA ATA TT -3’

ATF3-P3-F, 5’- GCT TTT GTG TTA ACC GGC GG -3’

ATF3-P3-R, 5’- GCG TGG TCA TTT TCT GGA GC -3’

ATF3-P4-F, 5’- GTG TGT GTC TCA GTG AGC GA -3’

ATF3-P4-R, 5’- TTA CTC CGT GTT GCC AGT CC -3’

ATF3-P5-F, 5’- TAA GCT TGG AAG TGG CGA GC -3’

ATF3-P5-R, 5’- TAT CTG CAC AAG TGG CTC CA -3’

ATF3-P6-F, 5’- GGG GAG AAA AGG AGC CAG TG -3’

ATF3-P6-R, 5’- AGA AAT GAA AAT GGG AGT GGG G -3’

TMEM27-P7-F, 5’- TCC TCC CAC CCT CTA ACC TT -3’

TMEM27-P7-R, 5’- GAC ATG CAC TTC CCA CCT TT -3’

TMEM27-P8-F, 5’- GAG AAT CGC AGA AGG GGT CG -3’

TMEM27-P8-R, 5’- CAG CCC ATC TCC TGT CAG TT -3’

SERPINB2-P9-F, 5’- CCT AAG CAT CGC TTG GGT CT -3’

SERPINB2-P9-R, 5’- GCT TTG GAC AAC CAT GGC CT -3’

SERPINB2-P10-F, 5’- GGG AGG GGC AAA GCT GTA TA -3’

SERPINB2-P10-R, 5’- CCC TCT ACA AAC ACC CCA CA -3’

Supplementary Figure 1: Analysis of SSX expression in a panel of melanoma cell lines. The expression of SSX family members was investigated in a panel of melanoma cell lines using Western blotting (A; with the E3 antibody recognizing SSX2-4) and quantitative PCR (B; with pan-SSX primers).

Supplementary figure 1

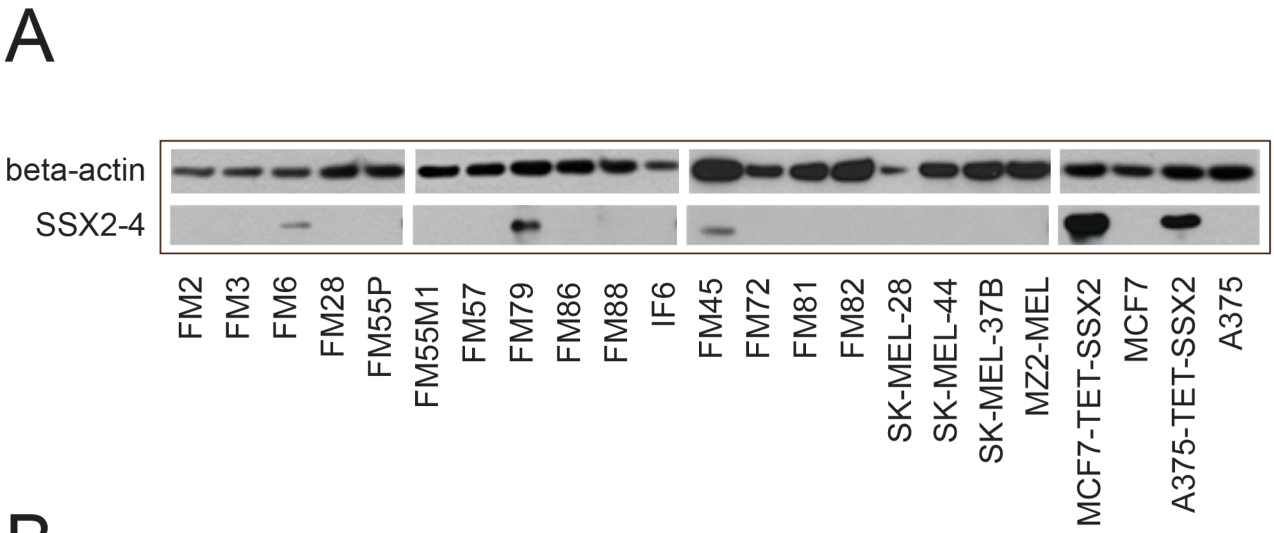

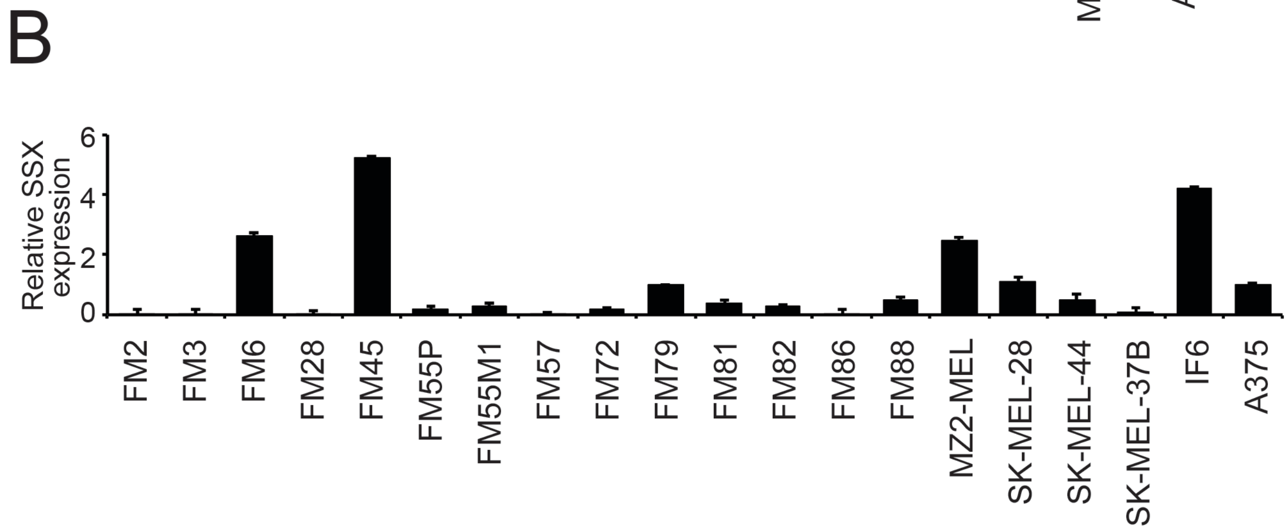

Supplementary Figure 2: Dot blot analysis of SSX2 expression in FM45 cells transduced with SSX2-specific shRNA lentiviral vectors (#20147 or #21692) or control (pLKO1). Lysates were spotted onto PVDF membrane and detection of SSX2 was carried out essentially as for Western Blotting (see materials and methods section).

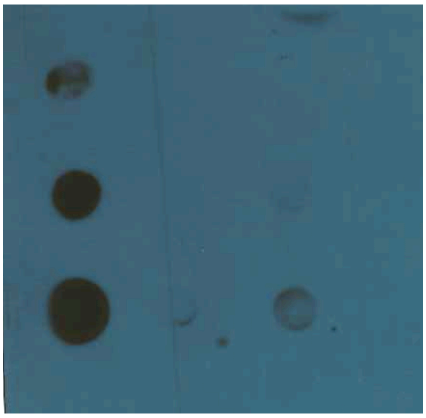

Supplementary Table 1: Global gene expression analysis of A375 with or without DOX-induced SSX2 expression. A375 cells with inducible DOX-inducible SSX2 expression (A375-TET-SSX2) were grown with or without DOX (50 ng/ml) for 48 hours and subjected to global gene expression analysis. Analysis was performed on 4 biological replicates for each group. Genes with FDR of less or equal to 0.05 and a fold change of at least 2 were classified as differentially expressed.

Column #	Column ID	gene_assignment	Gene Symbol	RefSeq	p-value(Do	p-value(SSX+do	Ratio(SSX+	Fold-Change
1247	7909271	NM_006850 // IL24 // interleukin 24 // 1q32 // 11009 /// NM_181339 // IL24 // in	IL24	NM_00685	2,00E-09	3,76E-09	8,6189	8,6189
12425	8021635	NM_001143818 // SERPINB2 // serpin peptidase inhibitor, clade B (ovalbumin), mem	SERPINB2	NM_00114	1,18E-08	1,12E-08	8,23878	8,23878
27379	8167251	---		---	1,50E-06	4,75E-07	4,25561	4,25561
21512	8112045	NM_007036 // ESM1 // endothelial cell-specific molecule 1 // 5q11.2 // 11082 ///	ESM1	NM_00703	1,05E-06	1,23E-06	3,76759	3,76759
12363	8021081	NM_001128588 // SLC14A1 // solute carrier family 14 (urea transporter), member 1	SLC14A1	NM_00112	4,91E-08	4,59E-08	3,64489	3,64489
27989	8172399	NM_174962 // SSX9 // synovial sarcoma, X breakpoint 9 // Xp11.23 // 280660 /// E	SSX9	NM_17496	2,88E-05	8,26E-06	3,45141	3,45141
4515	7942061	---		---	6,01E-07	1,01E-06	3,40276	3,40276
7484	7971565	NM_005767 // LPAR6 // lysophosphatidic acid receptor 6 // 13q14 // 10161 /// NM_	LPAR6	NM_00576	2,29E-07	9,78E-08	3,39923	3,39923
6466	7960947	NM_000014 // A2M // alpha-2-macroglobulin // 12p13.3-p12.3 // 2 /// ENST00000318	A2M	NM_00001	8,71E-07	1,17E-06	3,33393	3,33393
6803	7964631	NM_178539 // FAM19A2 // family with sequence similarity 19 (chemokine (C-C motif	FAM19A2	NM_17853	9,98E-07	1,51E-06	3,32966	3,32966
20367	8100798	NM_014465 // SULT1B1 // sulfotransferase family, cytosolic, 1B, member 1 // 4q13	SULT1B1	NM_01446	3,01E-06	1,41E-06	3,2419	3,2419
4213	7939314	NM_012153 // EHF // ets homologous factor // 11p12 // 26298 /// ENST00000257831	EHF	NM_01215	2,96E-07	1,57E-06	3,23021	3,23021
21528	8112198	NM_001017992 // ACTBL2 // actin, beta-like 2 // 5q11.2 // 345651 /// ENST0000042	ACTBL2	NM_00101	4,18E-07	1,60E-06	3,19028	3,19028
10211	7998927	---		---	4,06E-05	9,63E-05	2,99251	2,99251
5214	7948332	NM_004811 // LPXN // leupaxin // 11q12.1 // 9404 /// NM_001143995 // LPXN // leu	LPXN	NM_00481	1,83E-10	1,82E-10	2,98944	2,98944
19821	8095736	NM_001657 // AREG // amphiregulin // 4q13-q21 // 374 /// BC009799 // AREG // amp	AREG	NM_00165	8,53E-06	2,55E-06	2,95416	2,95416
12424	8021623	NM_003784 // SERPINB7 // serpin peptidase inhibitor, clade B (ovalbumin), member	SERPINB7	NM_00378	1,77E-05	0,000193187	2,86762	2,86762
14734	8044021	NM_016232 // IL1RL1 // interleukin 1 receptor-like 1 // 2q12 // 9173 /// NM_0038	IL1RL1	NM_01623	6,88E-06	2,96E-06	2,85482	2,85482
17435	8071420	NM_000185 // SERPIND1 // serpin peptidase inhibitor, clade D (heparin cofactor),	SERPIND1	NM_00018	8,29E-07	8,52E-07	2,65299	2,65299
15129	8047763	---		---	2,89E-05	1,71E-05	2,63006	2,63006
6784	7964460	NM_004083 // DDIT3 // DNA-damage-inducible transcript 3 // 12q13.1-q13.2 // 1649	DDIT3	NM_00408	3,98E-06	7,91E-06	2,60099	2,60099
1276	7909610	NM_001040619 // ATF3 // activating transcription factor 3 // 1q32.3 // 467 /// N	ATF3	NM_00104	5,38E-06	9,08E-06	2,59592	2,59592
5907	7954985	NM_032256 // TMEM117 // transmembrane protein 117 // 12q12 // 84216 /// ENST0000	TMEM117	NM_03225	3,43E-07	1,13E-06	2,5343	2,5343
25085	8145799	NM_080872 // UNC5D // unc-5 homolog D (C. elegans) // 8p12 // 137970 /// ENST000	UNC5D	NM_08087	7,63E-08	4,06E-08	2,52614	2,52614
11495	8012326	NM_021628 // ALOXE3 // arachidonate lipoxygenase 3 // 17p13.1 // 59344 /// ENST0	ALOXE3	NM_02162	1,80E-06	1,75E-06	2,47336	2,47336
18392	8081233	---		---	0,0058915	0,00292791	2,47178	2,47178
22512	8121257	NM_001198 // PRDM1 // PR domain containing 1, with ZNF domain // 6q21-q22.1 // 6	PRDM1	NM_00119	1,65E-07	6,12E-07	2,43068	2,43068
27291	8166469	NR_027783 // SAT1 // spermidine/spermine N1-acetyltransferase 1 // Xp22.1 // 630	SAT1	NR_027783	1,86E-06	3,03E-06	2,41167	2,41167
27891	8171472	NM_020665 // TMEM27 // transmembrane protein 27 // Xp22 // 57393 /// ENST0000038	TMEM27	NM_02066	1,20E-06	5,80E-06	2,37604	2,37604
25807	8151890	NM_033285 // TP53INP1 // tumor protein p53 inducible nuclear protein 1 // 8q22 /	TP53INP1	NM_03328	0,0001183	0,00010145	2,3562	2,3562
5847	7954330	NM_019844 // SLCO1B3 // solute carrier organic anion transporter family, member	SLCO1B3	NM_01984	9,32E-06	0,000113622	2,33707	2,33707
5213	7948330	NM_001005218 // OR5B21 // olfactory receptor, family 5, subfamily B, member 21 /	OR5B21	NM_00100	3,08E-06	3,49E-06	2,30435	2,30435
20819	8104901	NM_002185 // IL7R // interleukin 7 receptor // 5p13 // 3575 /// ENST00000303115	IL7R	NM_00218	1,84E-06	3,12E-06	2,28678	2,28678
28573	8177222	NM_013230 // CD24 // CD24 molecule // 6q21 // 100133941 /// ENST00000382840 // C	CD24	NM_01323	0,0001275	0,000330095	2,2426	2,2426
8039	7976826	NR_003219 // SNORD114-26 // small nucleolar RNA, C/D box 114-26 // 14q32 // 7676	SNORD114-26	NR_003219	4,65E-05	3,50E-05	2,20437	2,20437
22860	8124448	NM_003543 // HIST1H4H // histone cluster 1, H4h // 6p21.3 // 8365 /// BC120939 /	HIST1H4H	NM_00354	5,41E-07	7,23E-07	2,17235	2,17235
14779	8044532	NM_014439 // IL1F7 // interleukin 1 family, member 7 (zeta) // 2q12-q14.1 // 271	IL1F7	NM_01443	2,42E-05	1,88E-05	2,1702	2,1702
962	7906140	NM_199173 // BGLAP // bone gamma-carboxyglutamate (gla) protein // 1q25-q31 // 6	BGLAP	NM_19917	4,60E-06	9,54E-06	2,16493	2,16493
18452	8081818	---		---	0,0012616	0,00207233	2,14346	2,14346
18406	8081341	AK127584 // FAM172B // family with sequence similarity 172, member B pseudogene	FAM172B	AK127584	0,0003669	0,000319969	2,13689	2,13689
4203	7939173	NM_001077242 // DEPDC7 // DEP domain containing 7 // 11p13 // 91614 /// NM_13916	DEPDC7	NM_00107	1,03E-06	4,98E-06	2,13401	2,13401
11782	8015210	NM_033184 // KRTAP2-4 // keratin associated protein 2-4 // 17q12-q21 // 85294 //	KRTAP2-4	NM_03318	2,70E-07	8,19E-07	2,11261	2,11261
12192	8019576	NM_033184 // KRTAP2-4 // keratin associated protein 2-4 // 17q12-q21 // 85294 //	KRTAP2-4	NM_03318	2,70E-07	8,19E-07	2,11261	2,11261
28291	8175153	---		---	0,000149	0,000122335	2,11018	2,11018
19795	8095504	NM_001145006 // MUC7 // mucin 7, secreted // 4q13-q21 // 4589 /// NM_001145007 /	MUC7	NM_00114	4,83E-07	1,13E-06	2,08331	2,08331
16341	8060850	NM_001200 // BMP2 // bone morphogenetic protein 2 // 20p12 // 650 /// ENST000003	BMP2	NM_00120	8,40E-08	9,70E-08	2,08262	2,08262
6128	7957260	NM_006851 // GLIPR1 // GLI pathogenesis-related 1 // 12q21.2 // 11010 /// NM_007	GLIPR1	NM_00685	1,49E-05	1,86E-05	2,07659	2,07659
1033	7906786	NM_032738 // FCRLA // Fc receptor-like A // 1q23.3 // 84824 /// ENST00000236938	FCRLA	NM_03273	1,14E-05	1,41E-05	2,05701	2,05701
28094	8173444	NM_000206 // IL2RG // interleukin 2 receptor, gamma (severe combined immunodefic	IL2RG	NM_00020	4,19E-06	7,18E-06	2,05252	2,05252
2675	7923772	NM_001001552 // LEMD1 // LEM domain containing 1 // 1q32.1 // 93273 /// ENST0000	LEMD1	NM_00100	9,07E-05	0,000146743	2,0349	2,0349
1538	7912145	NM_001561 // TNFRSF9 // tumor necrosis factor receptor superfamily, member 9 //	TNFRSF9	NM_00156	7,75E-06	1,82E-05	2,02233	2,02233
7950	7976012	NM_004796 // NRXN3 // neurexin 3 // 14q31 // 9369 /// NM_001105250 // NRXN3 // n	NRXN3	NM_00479	3,52E-07	8,87E-07	2,02198	2,02198
7853	7974870	NM_003082 // SNAPC1 // small nuclear RNA activating complex, polypeptide 1, 43kD	SNAPC1	NM_00308	5,11E-05	7,65E-05	2,01987	2,01987
19392	8091678	NM_024621 // VEPH1 // ventricular zone expressed PH domain homolog 1 (zebrafish)	VEPH1	NM_02462	5,23E-05	0,000438276	2,01306	2,01306
18437	8081620	NM_013259 // TAGLN3 // transgelin 3 // 3q13.2 // 29114 /// NM_001008273 // TAGLN	TAGLN3	NM_01325	1,11E-06	7,46E-06	2,00057	2,00057
2282	7919715	NM_030920 // ANP32E // acidic (leucine-rich) nuclear phosphoprotein 32 family, m	ANP32E	NM_03092	6,61E-05	0,000286806	0,497279	-2,01094
24418	8139656	NM_001001555 // GRB10 // growth factor receptor-bound protein 10 // 7p12-p11.2 /	GRB10	NM_00100	2,13E-07	3,23E-07	0,494721	-2,02134
26898	8162472	NM_021570 // BARX1 // BARX homeobox 1 // 9q12 // 56033 /// ENST00000253968 // BA	BARX1	NM_02157	2,44E-05	5,26E-05	0,492098	-2,03211
11195	8008784	NM_018304 // PRR11 // proline rich 11 // 17q22 // 55771 /// ENST00000262293 // P	PRR11	NM_01830	0,0008658	0,000991861	0,492028	-2,03241
18849	8085754	NM_001012410 // SGOL1 // shugoshin-like 1 (S. pombe) // 3p24.3 // 151648 /// NM_	SGOL1	NM_00101	0,000544	0,00165914	0,482331	-2,07326
27159	8165345	NM_001606 // ABCA2 // ATP-binding cassette, sub-family A (ABC1), member 2 // 9q3	ABCA2	NM_00160	3,06E-07	7,26E-07	0,480048	-2,08312
3237	7929258	NM_004523 // KIF11 // kinesin family member 11 // 10q24.1 // 3832 /// ENST000002	KIF11	NM_00452	0,0011378	0,000872068	0,474954	-2,10547
5876	7954631	NM_018099 // FAR2 // fatty acyl CoA reductase 2 // 12p11.22 // 55711 /// ENST000	FAR2	NM_01809	5,74E-06	1,82E-05	0,473597	-2,1115
5273	7948900	NR_002561 // SNORD30 // small nucleolar RNA, C/D box 30 // 11q13 // 9299	SNORD30	NR_002561	0,0003737	0,000390954	0,465857	-2,14658
8681	7982358	NM_014783 // ARHGAP11A // Rho GTPase activating protein 11A // 15q13.2 // 9824 /	ARHGAP11A	NM_01478	0,0016013	0,00107898	0,465664	-2,14747
5276	7948906	NR_002563 // SNORD27 // small nucleolar RNA, C/D box 27 // 11q13 // 9301 /// AK0	SNORD27	NR_002563	0,0005477	0,000528738	0,463163	-2,15907
8253	7978846	NM_002692 // POLE2 // polymerase (DNA directed), epsilon 2 (p59 subunit) // 14q2	POLE2	NM_00269	2,26E-05	2,16E-05	0,461776	-2,16555
20681	8103728	NM_001130688 // HMGB2 // high-mobility group box 2 // 4q31 // 3148 /// NM_002129	HMGB2	NM_00113	2,24E-07	1,41E-07	0,456244	-2,19181
8709	7982663	NM_001211 // BUB1B // budding uninhibited by benzimidazoles 1 homolog beta (yeas	BUB1B	NM_00121	0,000121	7,90E-05	0,44784	-2,23294
25567	8149955	NM_018492 // PBK // PDZ binding kinase // 8p21.2 // 55872 /// ENST00000301905 //	PBK	NM_01849	2,73E-05	1,71E-05	0,439587	-2,27486
5271	7948896	NR_000008 // SNORD22 // small nucleolar RNA, C/D box 22 // 11q13 // 9304 /// AK0	SNORD22	NR_000008	0,0006841	0,00160394	0,434027	-2,30401
2626	7923189	NM_014875 // KIF14 // kinesin family member 14 // 1q32.1 // 9928 /// ENST0000036	KIF14	NM_01487	0,0006044	0,000393084	0,427659	-2,33831
8285	7979307	NM_014750 // DLGAP5 // discs, large (Drosophila) homolog-associated protein 5 //	DLGAP5	NM_01475	0,0007015	0,000494716	0,425525	-2,35004
5272	7948898	NR_002560 // SNORD31 // small nucleolar RNA, C/D box 31 // 11q13 // 9298 /// AK0	SNORD31	NR_002560	0,0005382	0,000730798	0,410952	-2,43337
15001	8046488	NM_031942 // CDCA7 // cell division cycle associated 7 // 2q31 // 83879 /// NM_1	CDCA7	NM_03194	1,65E-08	8,11E-09	0,408695	-2,44681
2615	7923086	NM_018136 // ASPM // asp (abnormal spindle) homolog, microcephaly associated (Dr	ASPM	NM_01813	2,35E-05	1,94E-05	0,386636	-2,58641
1046	7906930	NM_145697 // NUF2 // NUF2, NDC80 kinetochore complex component, homolog (S. cere	NUF2	NM_14569	8,49E-05	5,70E-05	0,379926	-2,63209
1284	7909708	NM_016343 // CENPF // centromere protein F, 350/400ka (mitosin) // 1q32-q41 // 1	CENPF	NM_01634	2,15E-05	3,30E-05	0,357998	-2,79331
